# Ssn6-Tup1 global transcriptional co-repressor: Role of the N-terminal glutamine-rich region of Ssn6

**DOI:** 10.1371/journal.pone.0186363

**Published:** 2017-10-20

**Authors:** Athanassios Tartas, Christoforos Zarkadas, Maria Palaiomylitou, Niki Gounalaki, Dimitris Tzamarias, Metaxia Vlassi

**Affiliations:** 1 Institute of Biosciences & Applications, National Centre for Scientific Research “Demokritos”, Ag. Paraskevi Attikis, Athens, Greece; 2 Institute of Molecular Biology & Biotechnology, Foundation for Research and Technology, Heraklion, Crete, Greece; 3 Biology Department, University of Crete, Heraklion, Crete, Greece; Russian Academy of Medical Sciences, RUSSIAN FEDERATION

## Abstract

The Ssn6-Tup1 complex is a general transcriptional co-repressor formed by the interaction of Ssn6, a tetratricopeptide repeat (TPR) protein, with the Tup1 repressor. We have previously shown that the N-terminal domain of Ssn6 comprising TPRs 1 to 3 is necessary and sufficient for this interaction and that TPR1 plays critical role. In a subsequent study, we provided evidence that in the absence of Tup1, TPR1 is susceptible to proteolysis and that conformational change(s) accompany the Ssn6-Tup1 complex formation. In this study, we address the question whether the N-terminal non-TPR, glutamine-rich tail of Ssn6 (NTpolyQ), plays any role in the Ssn6/Tup1 association. Our biochemical and yeast-two-hybrid data show that truncation/deletion of the NTpolyQ domain of Ssn6 results in its self association and prevents Tup1 interaction. These results combined with *in silico* modeling data imply a major role of the NTpolyQ tail of Ssn6 in regulating its interaction with Tup1.

## Introduction

The Ssn6-Tup1 co-repressor complex is consisted of two proteins, Ssn6 and Tup1 physically associated in a stoichiometry of 1:4 [1]. The Ssn6-Tup1 complex does not bind DNA directly but is recruited to target gene promoters *via* physical interactions with gene-specific DNA-binding repressor proteins under certain conditions [2–5]. Transcriptional repression by Ssn6-Tup1 is highly conserved across species, as proteins homologues to Ssn6 and Tup1 have been identified in terms of sequence similarity and functional homology in worms, flies, plants and mammals suggesting a universal molecular mechanism of transcriptional repression [2,6].

**Fig 1:**
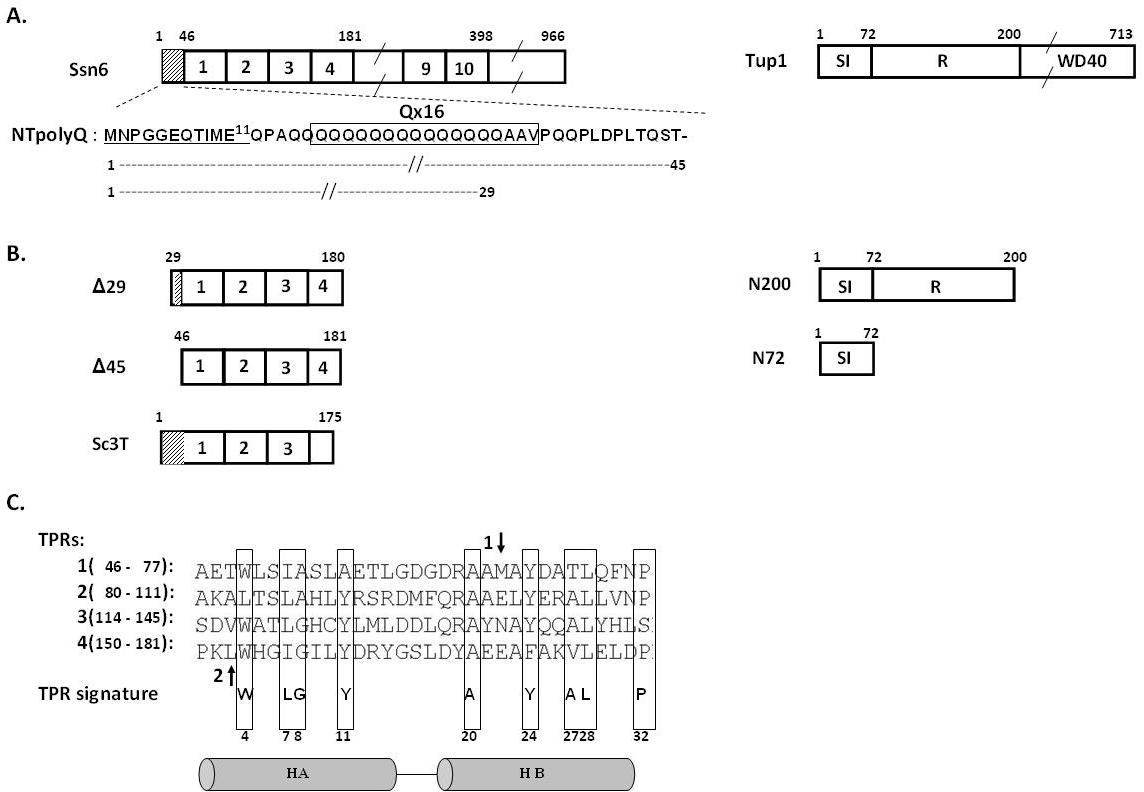
Ssn6 and Tup1 protein domains. (Α) Schematic representation of the Ssn6 and Tup1 protein architectures. Various sub-domains of both proteins (see text) are indicated. TPR repeats are illustrated as numbered boxes. (B) Schematic diagrams of Ssn6 (Left) and Tup1 (Right) fragments used in this study. (C) Sequence alignment of TPR repeats 1 to 4 of Ssn6. Predicted TPR α-helices (HA, HB) are depicted as cylinders below the sequence. Residues of the TPR signature [10] are boxed and amino acid preferences for each TPR-motif position are indicated below the aligned sequences. Arrows denote two susceptible to proteolysis sites of Ssn6, as described in detail elsewhere [11].

The molecular mechanisms by which Ssn6-Tup1 represses transcription have been studied quite extensively; early studies demonstrated that the repression function of the complex is mediated by Tup1 and more specifically by a distinct Tup1 repression domain (amino acids:72–200, Fig 1, [5]). More recent experimental data indicated that this Tup1 repression domain affects both the chromatin structure and the function of the basic transcription machinery [7–8]. Besides the repression domain, Tup1 comprises two additional functionally distinct domains; a Tup1 oligomerization and Ssn6-interaction domain localized at its N-terminus (amino acids: 1–72) and a WD40 repeats domain at the C-terminus (Fig 1A).The Ssn6-interaction (SI) and repression (R) domains of Tup1 along with Ssn6 provide sufficient repression action at most Ssn6-Tup1 regulated genes, as it has been described in detail previously [9].

The crystal structure of the SI domain of Tup1 revealed that Tup1 tetramerizes *via* coiled-coil interactions resulting in a novel antiparallel four-helix bundle fold [12]. On the other hand, the N-terminal domain of Ssn6 consists of 10 tandem repeats of a 34-residue motif known as tetratricopeptide repeat (TPR, Fig 1A and 1C). The TPR constitutes a degenerate recurrent sequence motif, which is known to mediate protein-protein interactions [13–14]. Indeed, it has been shown that TPRs 1 to 3 of Ssn6 associate with Tup1, whereas the recruitment of Ssn6-Tup1 to specific groups of target promoters is mediated by interactions of various combinations of TPR4 to TPR10 with distinct DNA-binding repressor proteins, as has been extensively described in [9]. The N-terminal domain of Ssn6 also contains a non-TPR glutamine-rich tail preceding the 10 tandem TPRs (Fig 1A), the function of which remains unknown.

The structure of the TPR motif has been studied in detail and it is now well established that each TPR motif is composed of a pair of antiparallel α-helices, termed helices A and B (see Fig 1C), which pack in a 3-stranded coiled-coil manner resulting in super-helical architectures [13–16]. Such structures result in the generation of two surfaces: a concave surface formed by helices Α of tandem TPRs and a convex surface formed by both A and B helices. The concave surface in many TPR proteins has been originally proposed [16], and then shown experimentally, to be involved in ligand binding [17–19]. Using the canonical TPR structure as template we have previously modelled the 3D-structure of the TPR1-3 domain of Ssn6 involved in Tup1 binding [20]. The model revealed that the formed concave surface of this domain of Ssn6 is of a more hydrophobic nature compared to other known TPR structures, suggesting a different mode of the Ssn6-Tup1 interaction [20]. More recent studies of other TPR proteins have indeed shown that the convex surface may also be involved in ligand binding [21–23]. In addition, individual TPR motifs and regions outside the TPR array have been also found to be implicated in TPR-mediated interactions including homo-oligomerization of TPR proteins [22, 24–25].

Our previous model of the Ssn6 TPR1-3 domain combined with mutagenesis data indicated that the structural integrity of TPR1, as well as its proper positioning relatively to TPR2 are essential for the Ssn6-Tup1 interaction, as described in detail in [20]. An independent mutagenesis work also supported the importance of the structural integrity of TPR1 for this particular interaction of Ssn6 [26]. In a more recent study however, we have shown that in the absence of Tup1, TPR1 is highly dynamic and susceptible to proteolysis, and that proper folding accompanies the Ssn6-Tup1 interaction [11].

In this study, we questioned whether the N-terminal non-TPR glutamine-rich tail of Ssn6 (NTpolyQ) plays any role in the Ssn6-Tup1 association. Our biochemical and *in vivo* experiments show that truncation/deletion of this domain results in self association of Ssn6 and inability for Tup1-interaction. Our data combined with *in silico* modeling imply a major role of the NTpolyQ tail of Ssn6 in regulating its interaction with Tup1 and transcriptional repression.

## Materials and methods

### Ssn6 and Tup1 expressing plasmids

The plasmid encoding the four N-terminal TPRs of Ssn6p (Δ45; amino acids 46–181) fused to the GST protein was constructed by inserting a PCR generated fragment between the *SmaI* and *EcoRI* sites of pGEX-2T (Amersham Biosciences Novagen) expression vector. The PCR product was generated using the 5’-CCCGGGCGGAAACTTGGCTCTCC-3’ and 5’-GAATTCTCAGTCCAATTCCAAAA-CTTTGGC-3’ forward and reverse primers, respectively and was subsequently purified and digested with *XmaI* and *EcoRI*. The *XmaI* site of the product was filled in with Klenow polymerase fragment prior to ligation with the vector. The sequence of the coding region was confirmed by nucleotide sequencing.

The LexA-Sc3T (N175) derivative of Ssn6 containing 175 amino-terminal residues was constructed by inserting a BamHI-BstxI Ssn6 fragment to the YCp91 vector (*TRP1-CEN*) which contains the *ADH1* promoter, SV40 NLS and HA1 epitope [9]. LexA-Ssn6 Δ29 was constructed by inserting a BamHI-BglII Ssn6 fragment from the isolated pACTII plasmid (Ssn6 Δ29-Gal4) in to BamHI-Asp718 (Klenow) sites of YCp91. The LexA-Ssn6 Δ45 hybrid was constructed by inserting the Δ45 PCR fragment (see above) to the SmaI-Asp718 (Klenow) sites of YCp91. LexA-Tup1 derivatives, N72 and N200, were also expressed using the YCp91 vector as described previously [5]. The Vp16 activation hybrids Tup1 N72-Vp16, Ssn6 Sc3T-Vp16 and Ssn6 Δ45-Vp1 were expressed using the YEp92 vector [5].

### Two-hybrid interaction and transcriptional repression assays

Yeast strains FT5 *(MATalpha*,*ura3-52*,*trp1-Δ63*,*his3-Δ200*,*leu2*::*PET56)* and the deletion derivative *tup1Δ*::*HIS3* have been described previously [5]. For two-hybrid assays, yeast cells were transformed with JK103 (2μ, *URA3*), a multicopy plasmid in which the *lacZ* gene is expressed by an artificial promoter with four lexop upstream from the *GALl* TATA element [27].

The *lacZ* constructs used in the LexA-dependent repression assay are derivatives of the plasmids pLGA312S and JK1621 integrated at the *URA3* locus [5]. pLGA312S drives LacZ expression from the *CYCI* promoter [28]. JK1621 is a derivative pLGA312S with four LexA binding sites upstream of the two UASs [4]. Yeast cells were grown in glucose media containing casamino acids until OD_600_~0.5.

For the two-hybrid screening, a Gal4pAD–yeast genomic library was used (A. Ramne & P.Sunnerhagen, *unpublished*).

### Ssn6 Δ45 expression and purification

The Ssn6 Δ45 fragment was expressed as a GST fusion protein in *Escherichia coli*. BL21(DE3) cells were grown at 37°C in LB medium containing ampicillin (80 μg/ml) to an OD_600_ of 0.6 and induced with 0.7 mM isopropyl-β-D-thiogalactopyranoside (IPTG) at 30°C for 3 h. The bacterial cell pellet was lysed by sonication in lysis buffer (20 mM Tris-HCl pH 8.0, 20 mM NaCl, 18% v/v glycerol, 0.2% v/v sarcozyl, 5 mM EDTA and 2 mM DTT) with 0.2 mg/ml lysozyme. Triton X-100 was added to a final concentration of 0.24% v/v and the lysate was centrifuged at 50,000g for 40 min at 4°C. The GST-Δ45 fusion protein was purified by affinity chromatography on a Glutathione Sepharose 4B (GSH) (Amersham Biosciences) affinity column equilibrated with lysis buffer. After extensive washing of the column the bound GST-Δ45 protein was digested with thrombin protease (at a molar ratio of 1:400) at 16°C, to remove the GST moiety. The reaction was stopped by the addition of benzamidine. The resulting purified protein was subsequently loaded on a Biogel P100 (Amersham Biosciences) gel-filtration column equilibrated with 20 mM phosphate buffer pH 8.0, 300 mM NaCl and 1 mM DTT.

### Molecular weight determination of Δ45

The molecular weight of Δ45 was determined by gel-filtration chromatography. The chromatographic separation on the Biogel P100 column was calibrated using the protein standards: bovine serum albumin (66 kDa), ovalbumin (45 kDa), carbonic anhydrase (29 kDa), cytochrome c (12.4 kDa), and potassium dicromate. Blue dextran was used to determine the void volume (V_0_) of the column. The relative molecular weight of the elution peak of Δ45 was then estimated using a standard curve obtained by plotting the logarithm of the molecular weight of the known proteins against K_av_ = (V_e_—V_0_)/(V_t_—V_0_), with V_t_ being the total bed volume.

### Circular dichroism

Far-UV (180–260 nm) circular dichroism experiments were performed on a JASCO J-715 spectropolarimeter. All scans were performed using a 0.1 mm path-length quartz cell. The CD signal of the buffer was subtracted from the CD signal of the protein. Conversion of the observed ellipticities (θ) to molar ellipticities, [θ] and deconvolution of the CD spectrum were made using the CDNN program [29– 30].

### 3D modeling

#### Construction of initial models

An initial 3D-model of Ssn6 Δ45 was constructed using the Swiss-pdb viewer program [31] and based on the known crystal structure of a designed canonical TPR array (PDB ID: 1NA0 [32]). The produced Δ45 model was subsequently used as initial structure for a 100 ns-long molecular dynamics (MD) simulation in explicit water. The Swiss-pdb viewer was also used to construct an initial 3D-model of the entire N-terminal region of Ssn6 up to 2.5 TPRs (NTpolyQ_TPR2.5) as follows: The polyQ stretch and its flanking NTpolyQ regions were modeled in α-helical and extended conformations, respectively. The former conformation was chosen because it has been proposed that wild-type polyQ stretches may serve protein interactions via coiled-coil interactions [33], whereas the latter was based on previous disorder/order predictions [11]. The TPR part on the other hand, was obtained from the Δ45 model with a slightly modified TPR3 HA helix to mimic a capping helix.

#### MD simulations

To overcome kinetic trapping problems, the initial NTpolyQ_TPR2.5 model was submitted to a set of long enough (230 ns) replica-temperature exchange MD (REMD) simulations [34] at four temperatures (300, 320, 340 and 360 K) using implicit solvation and a procedure similar to that used in [35]. The representative structures of the dominant clusters of conformations within the last 20 ns of the REMD replicas at the two lower temperatures (closer to the physiological temperature) were subsequently submitted to an additional 100 ns long classical (at a single temperature) MD simulation each, in explicit water, in order to test their stability in a more realistic environment. Next, the TPR3 of Ssn6 was added to each one of the REMD models to produce the respective models of the entire Tup1 interaction domain of Ssn6 (NTpolyQ_TPRs1-3). The stability of these models was also assessed by an additional set of 100 ns-long classical solvated MD simulations.

The MD simulations in explicit water were carried out at a single temperature (300 K) using periodic dodecahedron boxes of TIP3P water molecules [36] and MD parameters as in [37]. All MD simulations were performed using the GROMACS4 (v. 4.6.3) software package [38] on a 64-core Dell server.

#### Analysis of the MD simulations

Convergence of the MD trajectories was assessed by monitoring the backbone root-mean-square-deviation (rmsd) of the Cα atoms from the initial structure along the 100 ns solvated MD trajectories. Cluster analysis used the *g_cluster* module of GROMACS and a rmsd cut-off of 1 Å for two structures to be considered neighbors. The representatives (structures with the smallest average rmsd from all other structures of a cluster) of the most populated clusters of conformations in the last 10 ns of the second sets of solvated MD trajectories were subsequently optimized by the steepest descent energy minimization with flexible water, in order to obtain the final models of the Δ45 and NTpolyQ_TPR1-3 fragments. Structure averaging, root-mean-square atomic fluctuation calculations and estimation of corresponding temperature-factors (B-factor) were also carried out for the last 10 ns of the solvated 100 ns MD trajectories using related analysis tools of GROMACS and bash scripts we developed for this purpose. Related GROMACS tools were also used for the estimation of secondary structure elements along the MD trajectories. The VMD program [39] was employed for visualization of the trajectories, whereas molecular model illustrations were rendered using PyMOL.

## Results and discussion

### An Ssn6p derivative lacking the N-terminal non-TPR tail self-associates *in vitro*

We have previously performed a yeast two hybrid screening in order to identify yeast proteins interacting with the Ssn6 co-repressor protein [40]. We used as bait a LexA hybrid containing the TPR domain of Ssn6 and a library of random genomic fragments fused to the Gal4p activation domain (see “Materials & Methods”). Among several novel Ssn6-interacting proteins [40–43], a derivative of Ssn6 itself was isolated (Ssn6 Δ29; amino acids: 30 to 180), which contains three intact TPRs, but lacks part of the N-terminal Ssn6 sequence preceding the TPR domain (amino acids: 1–29, Fig 1A and 1B). The N-terminal tail of Ssn6 (NTpolyQ; amino acids: 1 to 45) contains a stretch of 16 consecutive glutamine residues (Qx16; amino acids: 15–30), 15 of which being missing from the randomly isolated Δ29 clone (Fig 1A and 1B). This two-hybrid interaction suggested that truncation/deletion of the NTpolyQ tail of Ssn6 may result in Ssn6 self-association.

**Fig 2:**
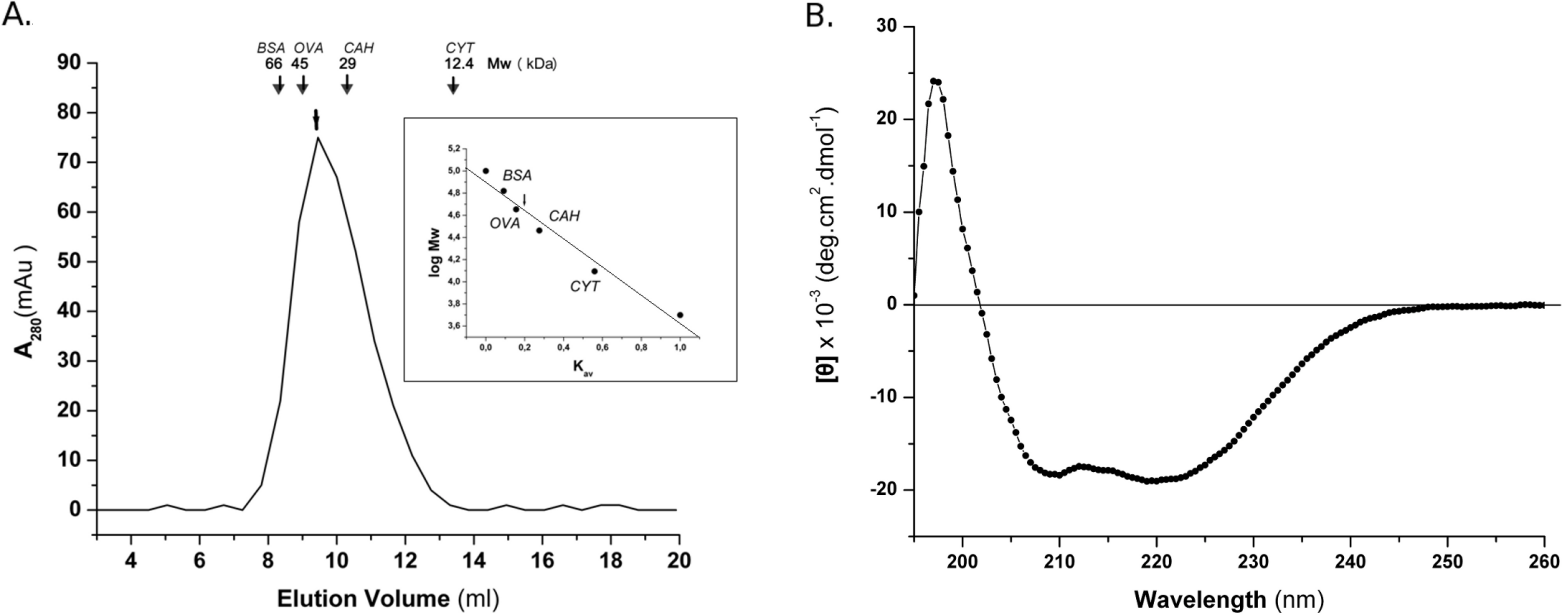
Biochemical characterization of Ssn6 Δ45. (A) Size exclusion chromatography: Elution profile of Δ45 from the Biogel P100 gel-filtration column relative to the protein standards: bovine serum albumin (*BSA)*, ovalbumin (*OVA)*, carbonic anhydrase (*CAH)* and cytochrom *c (CYT*). (Inset): The standard log M_w_ vs K_av_ curve used to estimate the relative molecular weight of Δ45. Δ45 is indicated by an arrow. (B) Circular dichroism: Far-UV CD spectrum of 20 μM of Δ45 recorded at 10°C in phosphate buffer (pH 8).

In order to investigate the above observation further, a fragment of Ssn6 essentially identical to the Δ29 fragment, but lacking the entire NTpolyQ tail (Ssn6 Δ45; amino acids: 46–181, Fig 1B), was expressed in recombinant form and purified biochemically as described in “Materials & Methods”. The molecular weight of the purified Ssn6 Δ45 fragment was estimated by size exclusion chromatography (SEC). Δ45 eluted from the SEC column at a volume equivalent to ~ 44 kDa (Fig 2A), which is approximately three times higher than the expected molecular mass of the monomeric form of this fragment (~ 15 kDa). This finding suggests that NTpolyQ-deleted Ssn6 derivatives self-associate, probably forming homo-trimeric species.

The conformation of this protein fragment was subsequently studied by circular dichroism (CD). The far-UV CD spectrum of Δ45 revealed a predominately α-helical structure as indicated by the characteristic double minima at 208 and 222 nm (Fig 2B). Deconvolution of the CD data gave an estimate of a high α-helical content (~60%). In addition, the [θ]_222_/[θ]_208_ ratio is >1 (Fig 2B), which is indicative of associated α-helices in a coiled-coil fashion [44–45], as expected for TPR structures. By contrast, an inversed [θ]_222_/[θ]_208_ ratio was observed in the CD spectrum of the Sc3T fragment of Ssn6, as described in detail in [11]. The CD data, in conjunction with the SEC results, suggest that the observed high molecular weight of Δ45 is not due to incorrect folding but it rather reflects self association of this Ssn6 fragment, in line with the yeast two hybrid results (see also below). Taken together our data so far suggest that the NTpolyQ tail of Ssn6 prevents oligomerization of this protein and may imply an important role of this domain in the Ssn6-Tup1 interaction.

### Ssn6 derivatives with either truncated or deleted NTpolyQ tail fail to interact with Tup1 and to repress transcription *in vivo*

We investigated the above hypothesis further by testing various Ssn6 and Tup1 derivatives (Fig 1B) for Ssn6-Ssn6 and Ssn6-Tup1 interactions using the yeast two-hybrid assay. As indicated in Table 1, LexA-Ssn6 Sc3T fails to interact with itself; indeed, when combined with Ssn6 Sc3T fused to the Vp16 activation domain, LexA-Ssn6 Sc3T activates transcription of the LexA-operator driven LacZ reporter at background levels, similarly to the LexA alone (Table 1). This result is in line with previous *in vitro* experiments performed by us and others demonstrating that either the full-length Ssn6 protein or the Sc3T fragment fail to self-associate and are clearly monomeric in solution [1,11]. However, this same LexA-Ssn6 Sc3T derivative strongly activates transcription when combined with the Ssn6 Δ29 derivative (lacking 29 amino acids of its NTpolyQ tail, Fig 1A and 1B) fused to the Gal4 activation domain (Table 1). Similarly, LexA-Ssn6 Sc3T activates transcription in combination with the Ssn6 Δ45 derivative (lacking the entire NTpolyQ tail (Fig 1B)) when the later is fused to the Vp16 activation domain (Table 1). LexA-Sc3T also activates transcription in combination with a Tup1 derivative (N72) comprising the minimal Ssn6-interaction (also indicated as SI in Fig 1) and Tup1 oligomerization region fused to the Vp16 activation domain (Tup1 N72-Vp16, Table 1). Conversely, LexA-Tup1 N72 activates transcription when combined with either Ssn6 Sc3T or Tup1-N72 when these derivatives are fused to Vp16 (Table 1). These findings are consistent with the observation that the Sc3T fragment of Ssn6 associates with Tup1, as described in detail elsewhere [5,11]. In contrast though, the LexA-Tup1 N72 hybrid does not interact with the Δ29 or the Δ45 Ssn6 derivatives (Table 1), indicating that NTpolyQ-deleted (either partially or completely) Ssn6 molecules fail to associate with Tup1.

**Table 1:** Two-hybrid assays for Ssn6-Ssn6 and Ssn6-Tup1 interactions.

	Activation hybrids			
LexA hybrids	Ssn6 Sc3T-Vp16	Ssn6 Δ29-Gal4	Ssn6 Δ45-Vp16	Tup1 N72-Vp16
LexA-Ssn6 Sc3T	4.5	93	122	51
LexA-Tup1 N72	80	4	3	56
LexA	4.1	4.5	4.2	3.2

Beta-galactosidase activities (average of at least three independent transformants) of wild-type strains expressing the indicated hybrid proteins. LexA hybrid proteins provide DNA-binding activity, while Gal4 and Vp16 activation domain hybrid proteins provide transcriptional activation activity. Yeast cells express the LacZ reporter by a minimal promoter containing four LexA operators followed by the *GAL1* TATA element and transcription initiation sequences. Values (Miller units) are normalized to OD_600_ of cells at the time of collecting and are accurate to +/-20%.

To investigate these observations further, we tested whether the Tup1-interaction defect of Ssn6 Δ29 and Δ45, possibly caused by their self-association property, would compromise their transcriptional repression activity *in vivo*. We employed a standard *in vivo* assay where various LexA hybrids were tested on a CYC1 reporter promoter containing four LexA operators (4 Lop) upstream from its natural enhancer elements. As shown in Table 2, LexA-Ssn6 Sc3T strongly repressed transcription from this promoter (4Lop) in a wild type strain while no effect was observed on the parental CYC1 promoter, which does not contain LexA operators (-Lop). In contrast, no repression effect was observed in a *tup1* strain, in agreement with previous results demonstrating that Ssn6 represses transcription by recruiting Tup1, which bears the repression activity of the co-repressor complex [5]. Consistently, LexA-Tup1 N200, which comprises the Tup1 repression domain, represses transcription in both wild type and *tup1* strains (Table 2). However, the LexA-Ssn6 Δ29 and LexA-Ssn6 Δ45 derivatives, which self associate and do not interact with Tup1 (shown above) fail to repress transcription; in fact they affect expression of the 4Lop-CYC1 LacZ reporter at the same extend as LexA alone (Table 2). It should be noted that this compromised repression activity could not be attributed to lower stability of Ssn6 Δ29 or Δ45 derivatives, as these same protein fragments displayed strong two hybrid interaction with Ssn6 Sc3T *in vivo* (see above, Table 1).

**Table 2:** *In vivo* repression by LexA-Ssn6 and LexA-Tup1 derivatives.

LexA hybrids	Fold repression		Promoter (strain)			
	(WT)	(*tup1*)	-Lop (WT)	4Lop (WT)	-Lop (*tup1*)	4Lop (*tup1*)
LexA-Ssn6 Sc3T	12	1.1	78	6.5	67	60
LexA-Tup1 N200	16	6	40	2.5	45	7.5
LexA-Ssn6 Δ29	1.0	1.0	75	76	84	80
LexA-Ssn6 Δ45	1.0	1.0	81	82	79	76
LexA	1.1	1.1	95	87	82	73

Beta-galactosidase activities (average of three independent transformants) of wild-type (WT), or *tupl* mutant yeast strains containing the indicated promoters and expressing the indicated proteins. *LacZ* values (Miller units) are normalized to OD_600_ of cells at the time of harvesting the yeast cultures and are accurate to +/30%. Fold repression represents the ratio of beta-galactosidase activities in strains containing plasmids that either express LacZ by the native CYC1 promoter (-Lop) or by a promoter derivative containing four overlapping LexA operators (4Lop) upstream of the *CYC1* promoter fused to the LacZ gene.

Combined, these data clearly suggest that the natural monomeric form of Ssn6, which can associate with Tup1 and repress transcription, depends on the integrity of its NTpolyQ tail preceding the TPR domain. Truncation or complete deletion of this N-terminal region results in Ssn6 self-association, inability for Tup1-interaction and subsequent loss of transcriptional repression activity *in vivo*. We thus hypothesize that the TPR region of Ssn6 alone has the tendency to self-associate and that the presence of the NTpolyQ tail prevents this association.

### Sequence analysis

**Fig 3:**
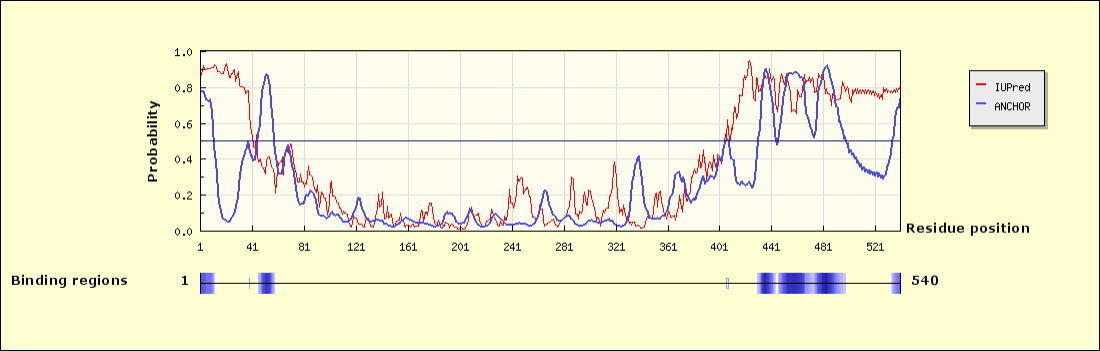
Prediction of disordered binding regions of Ssn6. Disorder/binding probability plot for the 540 N-terminal residues of Ssn6 as obtained using the ANCHOR server [46]. Predicted disordered/binding regions are depicted as blue shaded boxes underneath the probability plot.

In light of these new observations, the sequence of the N-terminal domain of Ssn6 was revisited. Analysis of the Ssn6 amino acid sequence using the ANCHOR method [46] predicted several disordered and potential binding regions, among which two are located in its Tup1 interaction domain; aa:1–11 and aa: 46–57 (Fig 3). The first region (N11) comprises the 11 N-terminal residues of the NTpolyQ tail (underlined in Fig 1A), whereas the second region coincides with the HA helix of TPR1 (Fig 1C). The latter observation is in line with the importance of TPR1 for Tup1 binding [20, 26] and consistent with our previous data showing that in the absence of Tup1, TPR1 is highly flexible and susceptible to proteolysis [11]. The first peptide (underlined sequence in Fig 1A), on the other hand, comprises two hydrophobic residues (Ile9, Met10) flanked by acidic residues (Glu6, Glu11) and its sequence resembles to that of binding sites of intrinsically disordered proteins (for a review, see [47]), such as the primary contact sites and the linear motifs. Such motifs also resemble to sequences recognized by TPR proteins like, for example, the Hsp70 IEEVD and Hsp90 MEEVD sequences that bind to TPR proteins, e.g. HOP and CHIP [18–19].

### 3D-modeling of Ssn6 fragments

To investigate this finding further, we applied *in silico* modeling techniques (homology modeling combined with molecular dynamics simulations; MD) on two Ssn6 fragments:

#### Δ45

**Fig 4:**
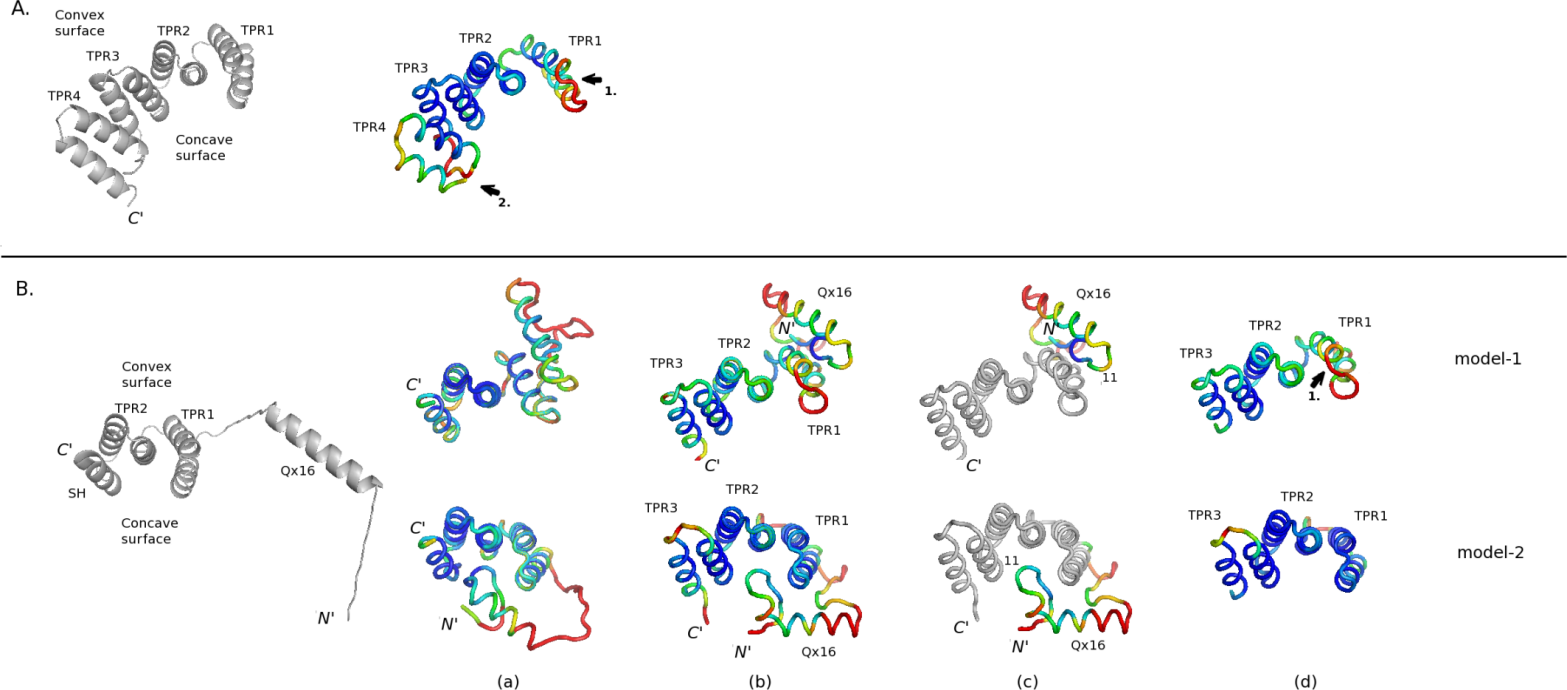
3D-modeling of Ssn6 fragments. (A) Modeling of Δ45: (Left) Cartoon representation of the initial 3D-model used for the explicit MD simulation. (Right) Average structure obtained from the last 10ns of the 100 ns MD trajectory, colored according to estimated atomic B-factors: from blue to red for low and high atomic fluctuations, respectively. (B) Modeling of NTpolyQ_TPR1-3: (Left) Cartoon representation of the initial 3D-model of a NTpolyQ_TPRs2.5 fragment used for the REMD simulations. (Right) (a) Average structures obtained from the last 10ns of the solvated 100 ns MD trajectories of the two REMD models (see “Materials & Methods”). Coloring is according to estimated B-factors, as in A. (b), (c) and (d) Average structures obtained from the last 10ns of the second set of solvated 100 ns MD trajectories of the two NTpolyQ_TPR1-3 models colored according to B-factor values for the entire NTpolyQ_TPR1-3, NTpolyQ and TPR domains, respectively, for clarity. Various domains discussed in the text are labeled. Arrows point to susceptible to proteolysis sites of Ssn6, as described in [11] (also shown in Fig 1C).

Firstly, using the canonical TPR structure as template we modeled the 3D-structure of the Ssn6 Δ45 fragment. The initial 3D-model (Fig 4A, left panel) was subsequently subjected to a 100 ns long MD simulation in explicit water. Analysis of the MD trajectory showed that the average crossing angles between the HA and HB helices ranged from 155.8° ± 12.3 (for TPR4) to 169.6° ± 4.7 (for TPR1), values that are comparable with literature data [48]. However, the packing between helices of adjacent TPRs showed lower crossing angle values, especially in the case of TPRs1 and 2 (147.2° ± 5.9). In addition, the average distance between the HA helices of the first three TPRs ranged from 12.7Å ± 0.4 (between TPRs2 and 3) to 13.7 Å ± 0.5 (between TPRs1 and 2). Interestingly, these distances are longer than those found in canonical 34-residue TPRs but rather comparable to average HAi: HAi+1 distances found in the case of kinesin light chain 2 (KLC2; PDB ID: 3CEQ [49]), a naturally occurring 42-residue-long TPR-like containing protein [48].

As indicated by low atomic fluctuations (low B-factor values: in blue in Fig 4), the TPR region remained rather stable during the last 10 ns of the MD simulation, with the exception of TPR1 and the loop connecting TPRs3 and 4 (Fig 4A, right panel). Consistent with our previous limited proteolysis data, these two highly mobile regions (red regions indicated with arrows in Fig 4) coincide with two susceptible to proteolysis sites of Ssn6, as described in [11] (also indicated by arrows in Fig 1C); namely, a site located at the beginning of HB of TPR1 (indicated by arrow 1 in Figs 1C and 4), and a second major chymotryptic site (indicated by arrow 2 in Figs 1C and 4) that removes the entire Tup1 interaction domain (corresponds to chymotryptic product 2 in [11]). Moreover, the structural stability of TPRs2 and 3 as indicated by their low atomic fluctuations (Fig 4, right panel), is also in agreement with our previous limited proteolysis results showing that potential chymotrypsin cut sites existing within TPR2 and TPR3 sequences were not recognized even in prolonged incubation times, as described in [11]. This is in line with our previous MD data on the TPR1-3 domain of Ssn6 that also suggested a higher mobility of TPR1 compared to TPRs2 and 3 [11]. Taken together, these observations further support the idea of a flexible structure for the Ssn6 TPR1 in agreement with the ANCHOR predictions (Fig 3).

#### Tup1 interaction domain (NTpolyQ_TPR1-3)

In order to obtain a 3D-model of the entire Tup1 interaction domain we next modeled the 3D-structure of the Ssn6 fragment comprising the NTpolyQ tail and expanding up to TPR3 (NTpolyQ_TPR1-3). This Ssn6 fragment was chosen because it comprises the minimal Tup1 interaction domain of Ssn6.

Initially, a 3D-model of an Ssn6 fragment extending almost to TPR4 (Fig 4B, left panel) was generated using REMD simulations [34] with implicit treatment of water, as described in “Materials & Methods”. This fragment was chosen in order to reduce the computational cost of REMD simulations. The dominant clusters of conformations within the last 20 ns of the REMD replicas at the two lower temperatures incorporated approximately 65% and 45% of the conformational ensembles, respectively. The stability of the representative structures of these two ensembles in a more realistic environment was subsequently tested by a 100 ns-long classical MD simulation, in explicit water, for each one of the two REMD models.

As shown in Fig 4B, the resulting models showed two possible yet distinct conformations of the NTpolyQ tail relatively to the Ssn6 TPR structure. Namely in model-1, the NTpolyQ tail folded against the convex surface of the formed Ssn6 TPR super-helix, whereas it was accommodated in its concave groove in the case of model-2 (Fig 4B(a)). These two binding modes of the NTpolyQ tail relative to the Ssn6 TPR structure were preserved in the presence of TPR3 and after an additional 100 ns-long MD simulation in explicit water for each one of the models, as shown in Fig 4B(b).

As indicated by the low atomic fluctuations (low B-factor values: in blue in Fig 4), the TPR region remained rather stable during the last 10 ns of the MD trajectories, with the exception of TPR1 in the case of model-1 (Fig 4B(d); upper panel), consistent with our previous limited proteolysis data [11] and similar to the MD results of the Δ45 fragment (compare Fig 4A and 4B(d)). On the other hand, as reflected by higher atomic B-factors, the NTpolyQ tail exhibited higher mobility in both models, with the exception of the N11 region especially in the case of model-1 (Fig 4(c)). These observations are in agreement with previous disorder predictions [11] and the ANCHOR results (Fig 3).

**Fig 5:**
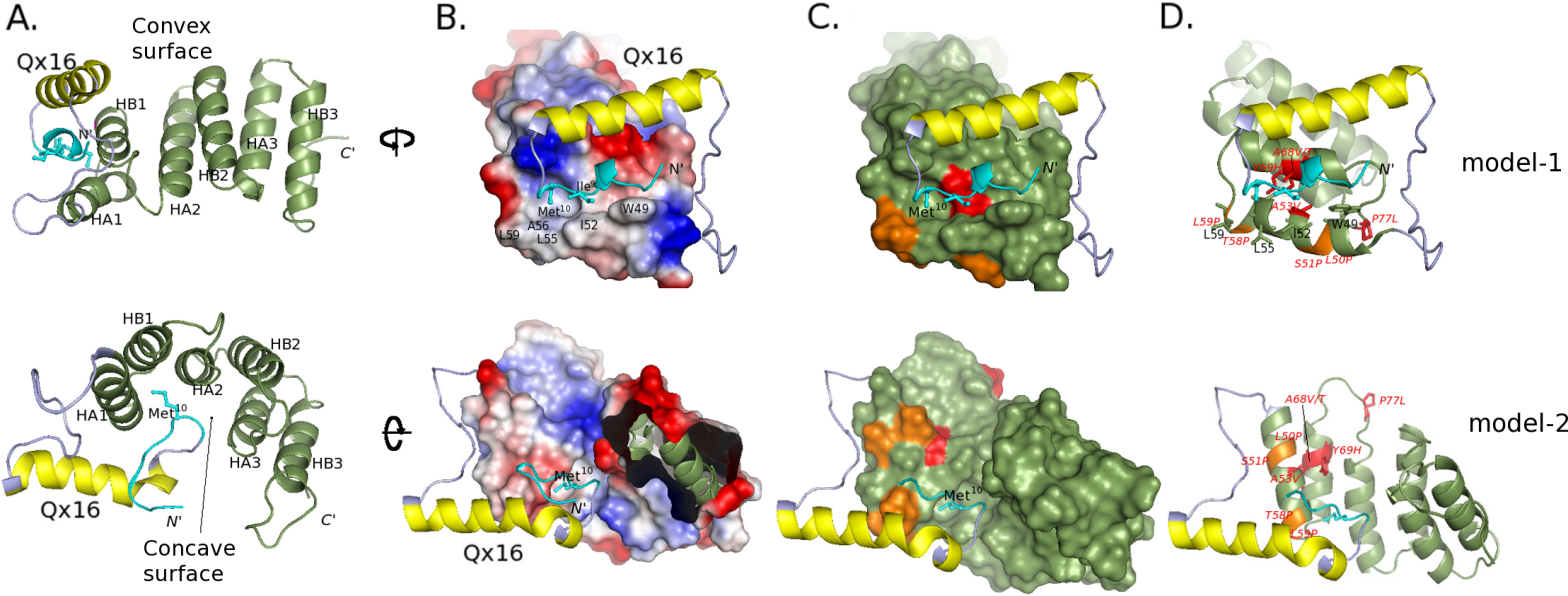
Details of the two final models of the NTpolyQ_TPR1-3 fragment. (A) The final models (Top: model-1; Bottom: model-2) in cartoon representation. The N11, Qx16 and TPR regions are colored in cyan, yellow and green, respectively. The TPR domain is also depicted as a surface colored according to (B) electrostatic potentials and (C) Ssn6 mutational data; in red/orange: point mutation sites disrupting/affecting Tup1 interaction [20, 26]. Hydrophobic Ssn6 residues are labeled in B. (D) Cartoon representation of the models with details of the mutations colored in C.

Of note however, is the observation that the Qx16 stretch despite its high mobility retained largely an α-helical conformation even throughout the second set of 100 ns MD trajectories of both models (in blue in S1 Fig). On the contrary, the regions of the NTpolyQ tail flanking the Qx16 stretch adopted various alternative structures, including random-coil, β-sheet and 3_10_-helix conformations (in white, red/black and grey, respectively in S1 Fig). For example, the 11-residue N11 region adopted 3_10_-helix (in model-1) and extended/random-coil conformations (in model-2) (S1 Fig), but was found to be anchored to the TPR super-helix in both models (Fig 5, in cyan). More precisely, the N11 fragment was packed against two distinct mainly hydrophobic pockets located at the convex and the concave surfaces in models-1 and -2, respectively (Fig 5). Both packing modes, although distinct, involve TPR1 (Fig 5) in agreement with the putative role of both the N11 and HA1 regions of Ssn6 as disordered/binding elements, also indicate by the ANCHOR predictions (Fig 3).

Collectively, our 3D-modeling data presented so far support the idea of transient alternative conformations of the NTpolyQ tail of Ssn6 resulting in temporary binding to its TPR domain in a manner reminiscent to that observed in fuzzy complexes [47]. This conformational plasticity seems to be facilitated by the intrinsic flexibility of the polyQ stretch.

Interestingly, as evidenced in Fig 5, Ssn6 point mutations that have been previously shown to both affect the TPR structure of Ssn6 and to disrupt Tup1-interaction [20, 26], map on the predicted NTpolyQ binding sites in both models of the NRTpolyQ_TPR1-3 fragment (Fig 5C and 5D). Given that TPRs1-3 alone are also capable to bind Tup1 [12], this observation implies that the NTpolyQ interactions modeled in here may mimic Tup1 binding. If this is the case, a displacement of the NTpolyQ tail must occur upon Tup1 binding and conformational change(s) must accompany the Ssn6-Tup1 complex formation. Indeed, our previous CD data have shown that conformational changes involving stabilization of a coiled-coil structure are coupled to binding of an N-terminal Tup1 fragment to the minimal Tup1-binding domain of Ssn6 (Sc3T fragment) [11].

### Comparison with known 3D-structures of other TPR proteins and TPR-mediated interactions

**Fig 6:**
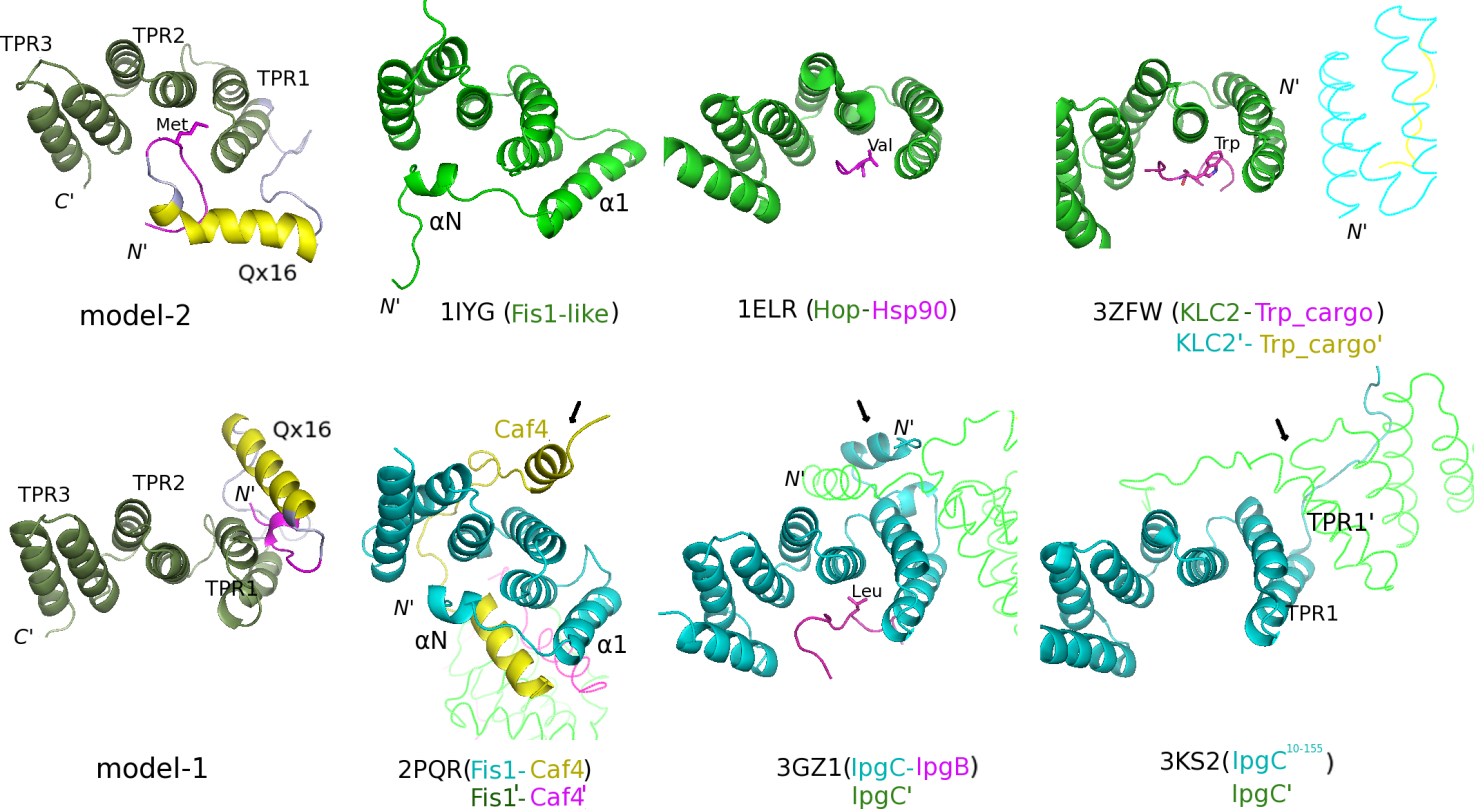
Comparison of the Ssn6 NTpolyQ_TPR1-3 models with known TPR structures/interactions from the literature. (Left) Cartoon representations of the final models shown in Fig 5 with a slightly different coloring: the N11 is colored in magenta, for comparison. The known 3D-structures from the literature shown in this figure correspond to the following PDB entries: 1IYG (solution structure of a Fis1-like protein from a mouse cDNA, unpublished); 1ELR (crystal structure of the TPR2 domain of human Hop in complex with an Hsp90 peptide [18]); 3ZFW (crystal structure of the TPR-like domain of KLC2 in complex with a cargo peptide [50]); 2PQR (crystal structure of yeast Fis1p in complex with a fragment of yeast Caf4p [22]); 3GZ1 (crystal structure of the IpgC chaperone from *Shigella flexneri* in complex with the chaperone binding region of IpgBp [24]) and 3KS2 (crystal structure of a truncated IpgC fragment showing an alternative head-to-head dimerization mode of this chaperone [25]). Molecules participating in dimer formation (obtained from symmetry-related molecules in the corresponding crystal structures) are depicted as ribbon models and indicated as accentuated words. Met10 of Ssn6 and important hydrophobic residues of linear ligand peptides in known TPR-mediated complexes are depicted in sticks and are labeled. Arrows indicate helical regions of the known crystal structures that are spatially similar to the Qx16 and N11 helices in model-1.

As already mentioned, TPR domains are versatile and accommodate different modes of protein recognition and/or oligomerization involving either or both their concave and convex surfaces. Non-TPR regions as well as individual TPR motifs have been found to contribute to such interactions. Some representative examples are shown collectively in Fig 6.

As evidenced in Fig 6, both binding modes of the NTpolyQ tail to the TPR super-helix of Ssn6, as suggested by our modeling data, comply with many TPR-mediated interactions derived from the literature: Of particular note is the case of the Fis1 protein, the N-terminal helix-turn-helix (αΝ-t-α1 in Fig 6) of which, is accommodated into the concave groove of its TPR-like region in a way reminiscent to that observed in model-2 (Fig 6), both in isolation (as in the unliganded yeast Fis1 protein [51] or in a Fis1-like hypothetical protein; PDB ID:1IYG, *unpublished*), as well as in the presence of peptides from its protein partners: the Mdv and Caf4 molecular adaptors (PDB IDs: 2PQN and 2PQR, respectively [22]). Interestingly, the yeast Fis1 uses its convex TPR surface to bind at the same time a second α-helix (indicated by an arrow in Fig 6) of Caf4 (PDB ID: 2PQR [22]). In addition, the manner by which the Ssn6 N11 region (magenta) of NTpolyQ in particular, is accommodated into the Ssn6 TPR1-3 concave groove in model-2, is reminiscent to that observed in complexes of TPR arrays with ligand peptides bound in extended conformations (e.g. Hop-Hsp90, PDB ID:1ELR [18]; IpgC-IpgB, PDB ID:3GZ1 [24]; KLC2 in complex with a tryptophan-acidic cargo peptide, PDB ID:3ZFW [50]) (Fig 6).

On the other hand, interactions involving TPR1 and the TPR convex surface, as in the case of our model-1, have been found to be implicated both in complex formation, as already mentioned, and in self-oligomerization of TPR proteins (see for example in Fig 6 the crystal structures of Fis1-Caf4 in 2PQR [22] and of two fragments of the IpgC chaperone in: 3GZ1 [24] and 3KS2 [25], respectively). More precisely, in model-1, the Qx16 α-helix and the N11 3_10_-helix (in magenta) packed against the HA/HB helices of TPR1 in a way spatially similar to that observed in various types of such convex-surface-involving TPR-mediated interactions (Fig 6; compare model-1 with α-helical regions pointed with arrows in known structures). Interestingly, the helical regions in these examples correspond to: an α-helix from the protein partner (Caf4 in 2PQR), N-terminal non-TPR helices of the TPR protein (IpgC in 3GZ1) or TPR helices of self associating TPR domains (IpgC^10-155^ in 3KS2, see also KLC2 in 3ZFW in Fig 6). In addition, the location of the Qx16 helix in model-2 is spatially similar to that of the non-TPR α1 helix of Fis1 protein, which has been found to be involved both in Caf4 binding and dimerization of the Fis1-Caf4 complex (Fig 6; compare model-2 with 2PQR). These observations imply an important role of the Qx16 track, most probably in preventing self-associations of the TPR domain of Ssn6 and/or in stabilizing its interaction with Tup1. Indeed, wild-type polyQ stretches have been proposed to play an important role in physiological protein-protein interactions most probably via coiled-coil associations [33].

Taken together, the above structural comparisons in conjunction with the *in vivo* data (Tables 1 and 2) further support the idea that the NTpolyQ tail of Ssn6 and Tup1 bind to overlapping (at least partially) regions of the Ssn6 TPR domain. This hypothesis is further supported by the fact that the sequence of the Ssn6 interaction domain of Tup1 resembles to that of the TPR-like domain of KLCs and that the Tup1 tetramer folds as a bundle of four α-helices through coiled-coil interactions between Tup1 dimers [12]. As already mentioned, KLCs are members of a family of proteins containing 42-residue TPR-like repeats, which are involved both in cargo binding and self-associations [48] (see also Fig 6; 3ZFW). Interestingly enough, we found a relatively high structural similarity between KLCs and a region of the Tup1 4-α-helix bundle comprising its Leu62 residue, which is essential for the interaction with Ssn6 [52] (*data not shown*).

## Conclusions

In this study we showed *in vitro* and *in vivo* that truncation/deletion of the N-terminal glutamine-rich tail of Ssn6 results in its self associations and that this “non-physiological” TPR-mediated oligomerization prevents the Ssn6-Tup1 interaction and its transcriptional repression activity. In addition, 3D-modeling suggested that the Ssn6 NTpolyQ tail is flexible and capable to adopt at least two alternative conformations relative to the TPR1-3 super-helix involving either the concave or the convex TPR surface. These alternative binding modes also engage TPR1 and most probably are facilitated by the intrinsic flexibility of the glutamine stretch of the NTpolyQ tail.

It is therefore tempting to speculate that, in the absence of Tup1, the NTpolyQ tail of Ssn6 through transient interactions with its TPR super-helix protects a non-physiological TPR1-mediated self association site of Ssn6, which at the same time serves its binding to Tup1. Alternatively, the NTpolyQ tail may stabilize the Ssn6 TPR structure and especially that of TPR1 (as observed in the case of model-2), which is essential for Tup1 interaction [20, 26]. However, such Ssn6 conformations must also be transient to comply with previous limited proteolysis data [11].

We propose that through such mechanism(s), the glutamine-rich tail of Ssn6 modulates its interaction with Tup1 and therefore its function.

## Supporting information

S1 Fig Secondary structure analysis of the NTpolyQ_TPR1-3 models. Monitoring of the secondary structure along the solvated 100 ns MD simulations of (Left) model-1 and (Right) model-2. The coloring of the secondary structure elements is as indicated in the bottom of the figure.
(TIF)
