# Supplementary figures and images for: Ssn6-Tup1 global transcriptional co-repressor: Role of the N-terminal glutamine-rich region of Ssn6
Source: PLoS One. 2017 Oct 20;12(10):e0186363. doi: 10.1371/journal.pone.0186363 (PMC5650148; doi:10.1371/journal.pone.0186363)

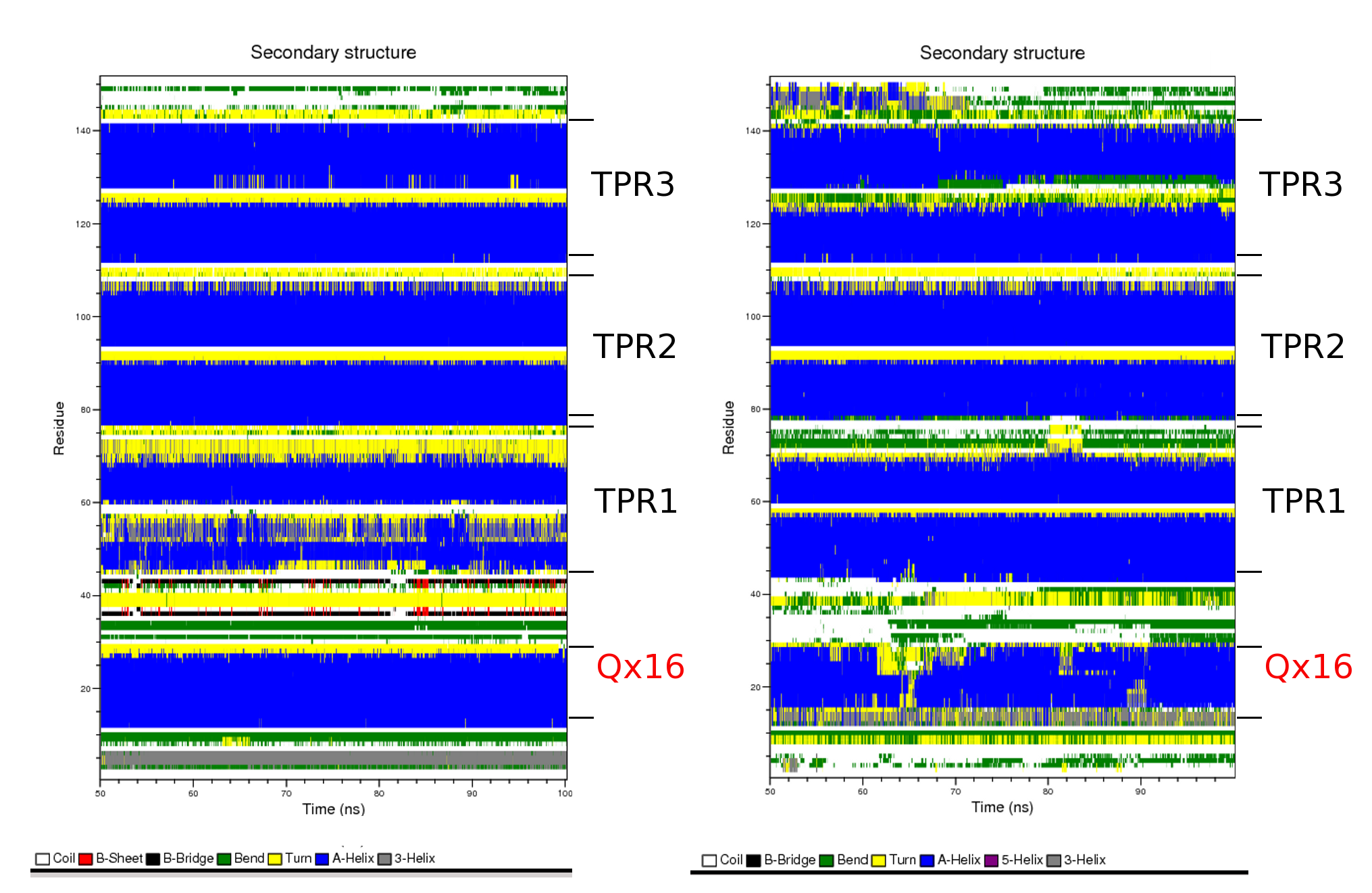

Supplement: S1 Fig — Monitoring of the secondary structure along the solvated 100 ns MD simulations of (Left) model-1 and (Right) model-2. The coloring of the secondary structure elements is as indicated in the bottom of the figure. (TIF) [file pone.0186363.s001.tif]
